# R-loop landscape in mature human sperm: Regulatory and evolutionary implications

**DOI:** 10.3389/fgene.2023.1069871

**Published:** 2023-04-17

**Authors:** Maurice Scheuren, Jonas Möhner, Hans Zischler

**Affiliations:** Division of Anthropology, Faculty of Biology, Institute of Organismic and Molecular Evolution, Johannes Gutenberg University Mainz, Mainz, Germany

**Keywords:** inherited gene regulation, primate evolution, retrotransposon, epigenetics, human sperm, R-loop

## Abstract

R-loops are three-stranded nucleic acid structures consisting of an RNA:DNA hybrid and a displaced DNA strand. While R-loops pose a potential threat to genome integrity, they constitute 5% of the human genome. The role of R-loops in transcriptional regulation, DNA replication, and chromatin signature is becoming increasingly clear. R-loops are associated with various histone modifications, suggesting that they may modulate chromatin accessibility. To potentially harness transcription-coupled repair mechanisms in the germline, nearly the entire genome is expressed during the early stages of male gametogenesis in mammals, providing ample opportunity for the formation of a transcriptome-dependent R-loop landscape in male germ cells. In this study, our data demonstrated the presence of R-loops in fully mature human and bonobo sperm heads and their partial correspondence to transcribed regions and chromatin structure, which is massively reorganized from mainly histone to mainly protamine-packed chromatin in mature sperm. The sperm R-loop landscape resembles characteristic patterns of somatic cells. Surprisingly, we detected R-loops in both residual histone and protamine-packed chromatin and localize them to still-active retroposons, ALUs and SINE-VNTR-ALUs (SVAs), the latter has recently arisen in hominoid primates. We detected both evolutionarily conserved and species-specific localizations. Comparing our DNA-RNA immunoprecipitation (DRIP) data with published DNA methylation and histone chromatin immunoprecipitation (ChIP) data, we hypothesize that R-loops epigenetically reduce methylation of SVAs. Strikingly, we observe a strong influence of R-loops on the transcriptomes of zygotes from early developmental stages before zygotic genome activation. Overall, these findings suggest that chromatin accessibility influenced by R-loops may represent a system of inherited gene regulation.

## 1 Introduction

Since the discovery of R-loops, one of the most abundant non-B-DNA structures in mammalian genomes (Al-Hadid and Yang, 2016), diverse types of biological functions and consequences have been characterized, most notably and recently their regulatory potential. Initially, R-loops were considered transcriptional byproducts that negatively affect genome stability by causing replication stress when they collide with the replication machinery (Aguilera and García-Muse, 2012). However, genomic results from antibody-based scans for the presence of R-loops suggest their occurrence in non-transcribed regions and consequently, various non-exclusive models for their biogenesis (Crossley et al., 2019; Sanz and Chédin, 2019). Current knowledge suggests that R-loops play key roles in transcriptional regulation and replication, genomic instability independent of replication stress, class switch recombination in B cells, and DNA damage and repair (Niehrs and Luke, 2020). In addition, R-loops modulate chromatin architecture through non-B-DNA structure and depletion of nucleosomes, becoming regulators of chromatin state and thus genes (Powell et al., 2013; Bayona-Feliu and Aguilera, 2021).

Recently, Adrian-Kalchhauser et al. (2020) proposed ‘*inherited gene regulation*’ as general feature of epigenetic inheritance. With R-loops potentially modulating gene expression in somatic cells, they could hypothetically act as intergenerationally epigenetic active elements when present in the germline. The persistence of R-loops in the genome of oocytes at metaphase II and in embryos at the preimplantation stage and at all cell cycles was documented by immunofluorescence. Maternal and paternal pronuclei showed the highest intensities at pronuclear stage 1. The authors hypothesize that the R-loops detected in the early pronuclear stages may be related to epigenetic mechanisms such as histone modifications and chromatin structure and may contribute to the genomic reprogramming that occurs in separate pronuclei during this early zygotic development, and put forward that the dynamics of the R-loop landscape are likely mechanistically linked to zygotic genome activation (ZGA) (Lee et al., 2022). This leaves open the question of whether R-loops occur in mature sperm cells and thus the search for their possible evolutionary consequences in the context of ‘non-genetic or epigenetic inheritance’ has not yet been addressed. To investigate a possible paternal contribution to epigenetic inheritance effective across evolutionary time scales, we focused on the male germline and examined sperm heads for the presence of R-loops. Sperm heads can be regarded as the precursors of the male pronucleus in the zygote because after the sperm has fertilized the egg, the decondensation of the sperm nucleus begins as the first step towards the formation of the male pronucleus (Lassalle and Testart, 1991). During spermiogenesis, testis-specific histone variants are incorporated into nucleosomes and subsequently hyperacetylated and removed, leading to the replacement of histones by transition proteins, and eventually to the transition to smaller protamines (Rathke et al., 2014; Bao and Bedford, 2016). This global remodeling together with DNA methylation and reprogramming of the genome could allow transcription and subsequent reintegration of transposable elements (TE), which mainly fall into two types of retroposons, namely, SINEs (short interspersed nuclear elements) and LINEs (long interspersed nuclear elements). This poses a threat to the integrity of the genome in the male germline at various stages of spermiogenesis (Soumillon et al., 2013; Ward et al., 2013). Although this histone-to-protamine transition is apparently incomplete and varies between different mammalian taxa (Torres-Flores and Hernández-Hernández, 2020), the resulting chromosome condensation is thought to coincide with gradual termination of transcription (D'Occhio et al., 2007; Rathke et al., 2007). In contrast, NGS data suggest that many different types of mRNA and non-coding RNA are present in fully differentiated sperm and can be transmitted intergenerationally into zygotes and early embryos (Wei et al., 2014; Santiago et al., 2021; Conine and Rando, 2022). Consistent with the histone-to-protamine transition, the defense mechanisms against the mobility of TEs, e.g., methylation of the source gene, are imperfect. In consequence this might lead to *de novo* integrations, especially of younger TEs, e.g. of ALUs, the most abundant hominoid SINE, which occurs in about 1/40 births (Schmid, 1991; Belyeu et al., 2021). When corrected for copy number, hominoid-specific SVA (SINE-VNTR-Alus) exhibit even greater mobility. The SVA is a composite of a (CCCTCT)_n_ hexamer repeat, two antisense ALU fragments, a VNTR and the env-gene plus 3′LTR from HERV-K10 (Cordaux and Batzer, 2009). SVAs are preferentially found in gene-rich regions and are of ever-increasing interest due to their co-evolution with TF and thus the potential regulation of expression in nearby genes (Savage et al., 2013; Gianfrancesco et al., 2019; Senft and Macfarlan, 2021; Barnada et al., 2022). SVAs are one of the youngest TEs in primates, and some copies are still active in the lineages that give rise to the extant primate representatives, bonobos and humans (Wang et al., 2005). Interestingly, the SVAs showed strong hypomethylation in human spermatozoa and are reported to be overrepresented in somatic R-loops (Molaro et al., 2011; Zeng et al., 2021).

In this study, we correlated both sperm head transcript profiles and supposedly transcriptionally inactive protamine-covered regions with R-loops (D'Occhio et al., 2007; Rathke et al., 2007), with transcription during spermatogenesis as main source for R-loop biogenesis. We could furthermore highlight the role of hominoid-specific, still actively transposing SVA, in R-loop formation in both humans (referred to as Hsa in figures and tables) and *Pan paniscus* (referred to as bonobo in the text; Ppa in figures and tables) sperm heads and demonstrate that R-loops possess the potential to represent a system of ‘*inherited gene regulation’* (Adrian-Kalchhauser et al., 2020).

## 2 Material and methods

### 2.1 Sperm head preparation

All ejaculate samples were non-invasively obtained. Human samples were provided by volunteers by masturbation after 2–3 days of sexual abstinence, with informed consent. The bonobo sample was obtained at Zoo Wuppertal. The sample was collected opportunistically (from the cage floor after the animal masturbated) and shipped at ambient temperature. All samples were stored at −25°C/-80°C until further processing.

The total ejaculate was centrifuged (16,000 x g, 5 min at room temperature) and the supernatant was discarded. The cell pellet was resuspended in lysis buffer (10 mM TRIS pH 8; 10 mM EDTA; 100 mM NaCl; 4% SDS). The suspension was centrifuged, the supernatant discarded and the previous step repeated. The pellet was resuspended in 1.35 mL lysis buffer, 150 µL 1 M dithiothreitol (DTT) was added for optimal sperm lysis, and the lysate was incubated at 55°C for 30 min.

### 2.2 DNA-RNA immunoprecipitation (DRIP) assay

DRIP was performed as previously described with minor modifications (Halász et al., 2017; Bou-Nader et al., 2022). Briefly, SureBeads™ Protein A Magnetic Beads (BioRad, United States) were pre-blocked with PBS containing 0.5% BSA and 5 mM EDTA and subsequently washed with wash buffer (PBS; 1% Triton X-100; 1 mM EDTA). To pre-immobilize the S9.6 antibody (Active Motif, United States), 100 µL pre-blocked beads were incubated with 5 µg of S9.6 antibody in binding buffer (50 mM TRIS; 0.14 M NaCl; 5 mM EDTA; 1% Triton X-100) at 4 °C for 2 h with rotation. DNA was isolated from sperm head pellets, lysed with DTT, using phenol/chloroform/isoamyl alcohol (25:24:1), precipitated with ethanol and resuspended in TE buffer. The MboI digested DNA was added to the beads and incubated overnight at 4°C. Bound beads were recovered and washed three times with wash buffer (PBS; 1% Triton X-100; 1 mM EDTA). Precipitates were eluted in elution buffer (10 mM Tris pH 8; 1 mM EDTA; 1% SDS) and 5 µL proteinase K (10 mg/mL) in 100 µL for 15 min at 65 °C. DNA was purified using a QIAquick^®^ PCR purification Kit (QIAGEN, Netherlands). Libraries were prepared by Novogene using the NEB Next Ultra DNA Library Prep Kit and sequenced on Illumina NovaSeq 6000 PE150 (Hsa n = 2, Ppa n = 1).

### 2.3 Bioinformatic methodology of the DRIP-seq analysis

DRIP-seq reads were aligned to GRCh38 genome and Mhudiblu_PPA_v0 respectively, using STAR (version 2.7.9a) with parameters according to Teissandier et al. (2019) for a TE-sensitive mapping (Teissandier et al., 2019), PCR-duplicated reads were removed using Picard (‘Picard Toolkit.’ 2019. Broad Institute, GitHub Repository. https://broadinstitute.github.io/picard/; Broad Institute) and converted using SAMTools (version 1.10). To assign TE-derived reads to individual peaks, the CSEM workflow (Chung et al., 2011) was used, and MACS (version 3.0.0a7) with parameters according to Zeng et al. (2021) was used for peakcalling with digested gDNA Input as control. We selected the output file ‘. broadPeak’ for the final peak detection results. Blacklisted regions of hg38 (obtained from http://mitra.stanford.edu/kundaje/akundaje/release/blacklists/hg38-human/hg38.blacklist.bed.gz) were removed using BEDTools (version 2.30.0) (Quinlan and Hall, 2010). Subsequently, the identified peaks were annotated using ‘annotatePeaks.pl’ from the Homer software (version 4.11) with hg38 for the human sample and a customized reference for bonobo respectively (Heinz et al., 2010). The customized reference was built from RefSeq (O'Leary et al., 2016) and RepeatMasker (Smit et al., 2008) datasets. Metaplots were created using R package ChIPseeker (Yu et al., 2015; Wang et al., 2022) and Venn diagrams were created using Intervene (version 0.6.1) (Khan and Mathelier, 2017).

### 2.4 RNA-seq and bioinformatic analysis

Total RNA was isolated from prepared sperm heads using the standard TRIzol-based protocol (Thermo Fisher Scientific, United States), followed by poly-A enrichment and sequencing by BGI Biotechnology using BGISEQ-500 Transcriptome PE100 (n = 1). RNA-seq reads were aligned to GRCh38 transcriptome using STAR (version 2.7.9a) with ‘--quantMode TranscriptomeSAM’. The RSEM Workflow (Li and Dewey, 2011) was used to calculate fragments per kilobase of transcript per million fragments mapped (FPKM) data, and transcripts with an FPKM <1 were discarded. RSeQC (version 5.0.1) was used for calculation of transcript integrity number (TIN), transcripts with TIN >75% were considered to be intact (Wang et al., 2012; Wang et al., 2016). GeneIDs of transcripts were used for annotation with Ensembl Release 108 (Cunningham et al., 2022).

### 2.5 Identification of genomic regions with GC skew

To define the regions displaying a GC skew in the human and bonobo genomes, we applied the SkewR pipeline 1.00 b using the most stringent model for GRCh38 and Mhudiblu_PPA_v0 respectively (Ginno et al., 2012; Ginno et al., 2013).

### 2.6 Gene ontology enrichment analysis

Gene ontology (GO) Biological Process 2021 enrichments were analyzed by using Enrichr (https://maayanlab.cloud/Enrichr/) (Chen et al., 2013; Kuleshov et al., 2016; Xie et al., 2021).

### 2.7 Motif analysis

The Simple Enrichment Analysis software (version 5.5.0) from MEME Suite (http://meme-suite.org/) was used with default settings to analyze the motifs present in R-loops (Bailey and Grant, 2021).

### 2.8 Retrieval of public datasets

We obtained the datasets (GSE57095, GSE40195, GSE144283 and GSE30340) from the GEO database (Table 1). If necessary, data was converted to GRCh38 using Liftover. We conducted the authors’ workflow for datasets PRJNA715579 and GSE44183 (Table 1).

**TABLE 1:** The information of the used datasets.

Dataset	Library type	Explanation	Species	Edit	References
GSE57095	ChIP-Seq	Residual histones H3K4me1, H3K27ac, H3.3	*Homo sapiens*	Liftover to GRCh38	Hammoud et al. (2014)
GSE40195	ChIP-Seq	Residual histones H3K14ac	*Homo sapiens*	Liftover to GRCh38	N/A
PRJNA715579	ChIP-Seq	Residual histones TH2B	*Homo sapiens*	Liftover to GRCh38	Patankar et al. (2021)
GSE144283	ChIP-Seq	H3S10p marks in IMR-5 cells	*Homo sapiens*	Liftover to GRCh38	Roeschert et al. (2021)
GSE44183	RNA-Seq	RNA expression in early human embryos	*Homo sapiens*		Xue et al. (2013)
GSE30340	Bisulfite-Seq	Methylome of mature human sperm	*Homo sapiens*	Liftover to GRCh38	Molaro et al. (2011)
PRJNA890147	DRIP-Seq	R-loop landscape of mature human sperm	*Homo sapiens*		This study
PRJNA890147	DRIP-Seq	R-loop landscape of mature chimp sperm	*Pan paniscus*		This study
PRJNA890147	RNA-Seq	Transcriptome of mature human sperm	*Homo sapiens*		This study

## 3 Results

### 3.1 R-loops as revealed by DRIP overlap with RNA-seq profiles in human sperm heads

**FIGURE 1:**
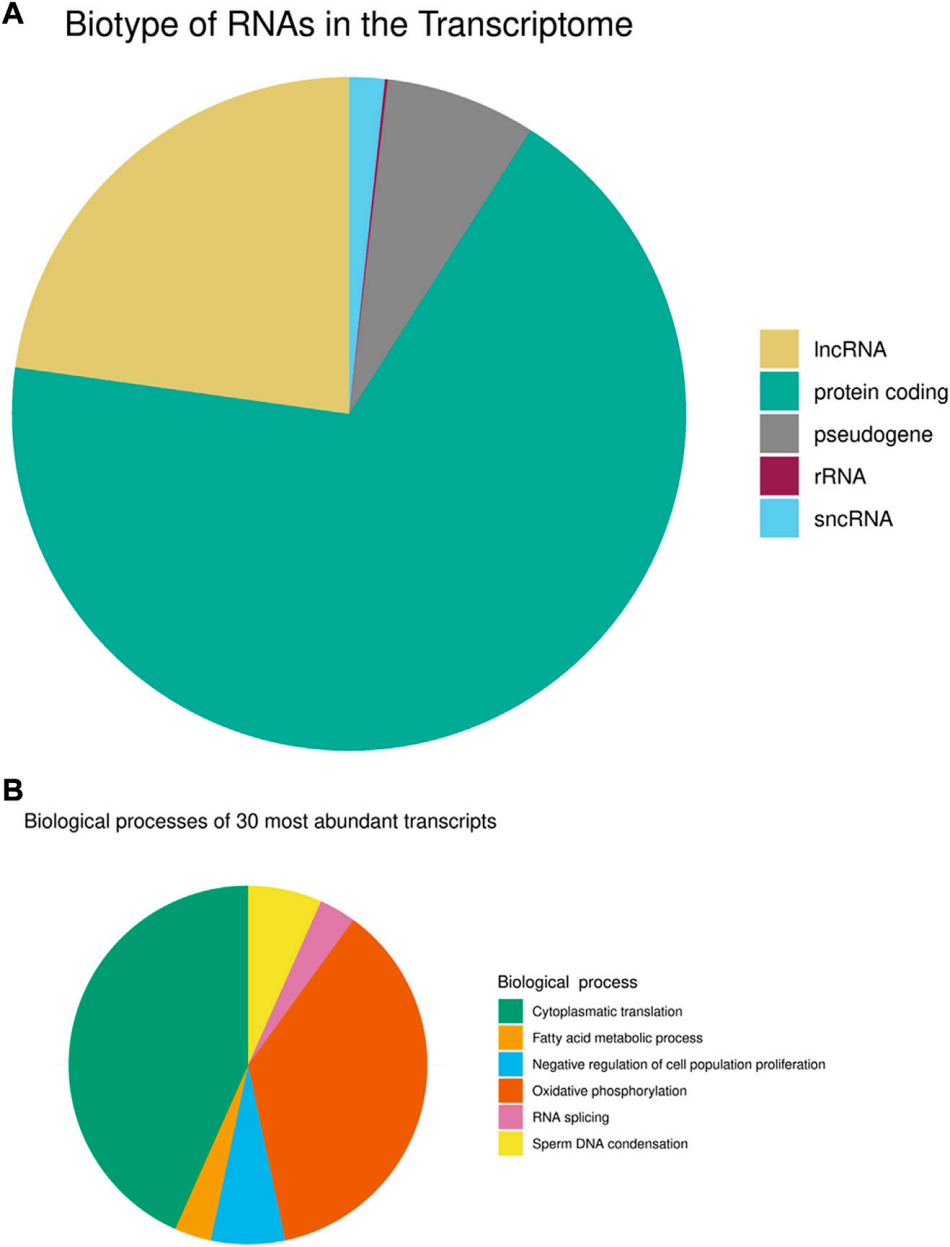
Transcriptome of mature human sperm **(A)** Biotype annotation of transcripts. **(B)** Biological processes of the thirty most abundant intact transcripts (TIN >75%).

Since sperm heads best reflect the situation in the male pronucleus of the zygote, it is important to determine the R-loops and transcripts localized in the sperm head. Moreover, the respective localization suggests that this paternal information could be contributed to the oocytes during fertilization (Peng et al., 2012). Therefore, we isolated sperm heads by applying the standard protocol for differential extraction of sperm heads, first in the absence of DTT as a reducing agent with repeated intermittent centrifugation and subsequent lysis of sperm heads in the presence of DTT (Jankova et al., 2019). The mature sperm transcriptome is either derived from residual RNA transcripts generated during the early stages of spermatogenesis or is produced through active transcription (Ren et al., 2017). Regardless of the transcript origin, the existing RNA can interact with sperm chromatin in a variety of ways and can be introduced into the oocyte as a substantial paternal contribution of diverse populations of RNAs (Sharma et al., 2018; Li and Klungland, 2020; Kretschmer and Gapp, 2022). Thus, the transcriptome is a major key for understanding R-loops in sperm. Therefore, we first investigated the overlap between human sperm head nascent transcripts and corresponding R-loops. To this end RNA was isolated from sperm heads lysed with DTT according to standard TRIZOL-based protocols with subsequent precipitation and subjected to deep sequencing with 45,811,118 clean reads obtained from RNA-seq. Applying bioinformatic routines as implemented in STAR, we detected and mapped 32,534 expressed protein-coding and RNA genes in the sperm transcriptome (genes with FPKM >1 were considered to be expressed). The annotated transcript population mainly consists of mRNAs and long non-coding (lnc)RNAs, followed by smaller fractions of transcribed pseudogenes and source transcripts of diverse small non-coding RNAs (Figure 1A; Supplementary Table S1). The complex mRNA, lncRNA and pseudogene landscape results from the global transcription during spermatogenesis. All thirty most abundant and intact (TIN >75%) transcripts are mRNAs and their translated proteins correspond mainly to the biological processes of oxidative phosphorylation, sperm DNA condensation and cytoplasmic translation (Figure 1B).

**FIGURE 2:**
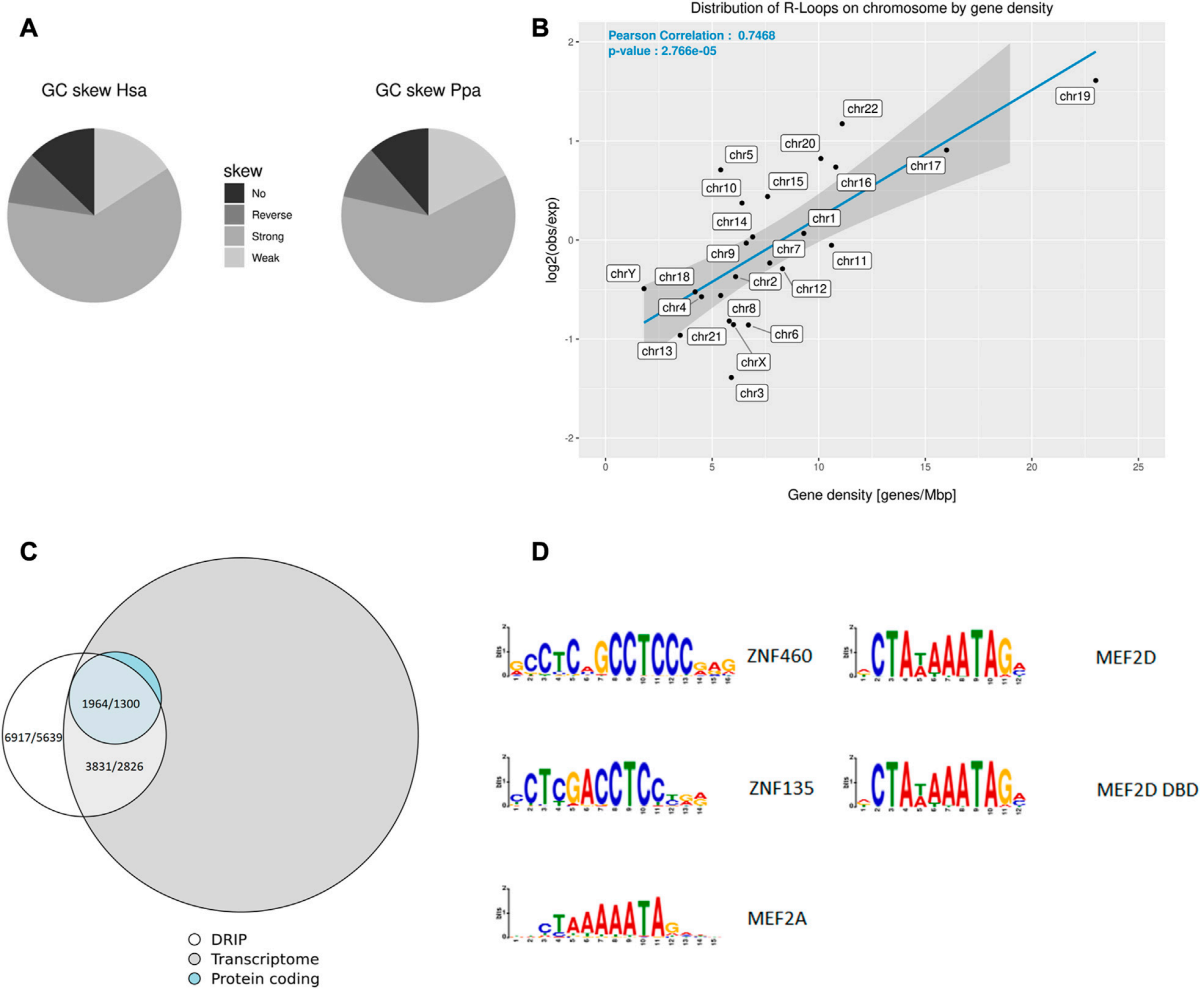
R-loop Distribution across the sperm genome and transcriptome. **(A)** Pie charts showing the distribution of human and bonobo genes with respect to their GC skew according to SkewR. **(B)** Relative proportion of R-loop sequences per chromosome compared to the gene density of the corresponding chromosome. **(C)** Venn diagrams showing the overlap between the human protein-coding transcriptome and R-loops, indicated are values for each replicate. **(D)** Most enriched motifs in R-loops in human and bonobo.

Next, we isolated sperm heads from the ejaculates and lysed them by adding DTT to a final concentration of 100 mM. Subsequently phenol-chloroform extraction and DNA isolation by precipitation were used to prepare an antibody-based ChIP-like genome-wide analysis of R-loops (DNA-RNA-immunoprecipitation, DRIP). To scrutinize the possible conservation of R-loop profiles in closely related primate representatives, we isolated sperm heads from a bonobo ejaculate and DRIP - analyzed the sample as well. Using DRIP-Seq and evaluating the data with STAR-mapping and CSEM to incorporate repetitive DNA regions into peaks annotation we detected 6278 ± 639 peaks covering 4.94 ± 1.37 Mbp of the human sperm genome of which 52.76% ± 2.64% were located in genes. The average GC content of R-loops was 54.99% ± 0.02%. To further investigate the correlation between R-loops and the base composition of the corresponding genomic region, we checked for GC skews in genes associated with R-loops (Table 2). The annotation by SkewR revealed that more than 75% of genes associated with R-loops showed a GC skew, which can facilitate the formation of R-loops (Figure 2A). Furthermore, the density of R-loops per chromosome strongly correlates (Pearson’s r = 0.7468, *p* < 0.001) with the chromosome specific gene density (Figure 2B). Both the strong GC skew and the gene dependency resembles the R-loops landscape of somatic cells, indicating transcription as the main contributor to R-loop formation. Because transcription is mostly inactivated in mature sperm, transcription during spermatogenesis and residual transcripts could facilitate R-loop formation. Therefore, we investigated the genome-wide relationship between the transcriptome of mature sperm and the R-loop landscape. We checked for overlaps between the two features and found that more than 60% of genomic R-loops had corresponding transcripts in the sperm head (*p* < 0.001, Hypergeometric test). Regarding the correlation between source genes of the transcripts and R-loops, we detected a strong bias towards protein-coding genes, in which 89,14 ± 0.07% were associated with R-loops, contrasting just 31,83 ± 10,78% of ncRNA genes (Figure 2C).

**TABLE 2:** Basic analysis of the DRIP-Seq experiments. Error shows ±standard error of the mean (SEM) among the biological replicates.

	Number of peaks	Coverage of peaks [Mbp]	Average GC content [%]	R-Loops associated with genes [%]
R-loops Hsa	6278 ± 639	4.94 ± 1.37	54.99 ± 0.02	52.76 ± 2.64
R-loops Ppa	17,560	6.31	51.55	60.18

To further investigate the contribution of transcription to the R-loop landscape, we used *Simple Enrichment Analysis* for a motif enrichment analysis and detected motifs for the transcription factors (TF) ZNF460 and ZNF135 in more than 20% of human R-loops (Figure 2D; Supplementary Table S2). Both, ZNF460 and ZNF135, function as TF for RNA polymerase II. RNA polymerase II is mainly responsible for the transcription of mRNAs, micro-RNAs (miRNAs) and small nuclear RNAs (snRNAs). Both motifs were also enriched in the R-loop landscape of the bonobo. Motifs of two closely related TF MEF2A and MEF2D, which are able to form a heterodimer, were enriched in human R-loops, too. The MEF2A motif was also more abundant in R-loops of the bonobo. The motif enrichment and R-loop bias towards protein-coding genes reinforces transcription during the spermatogenesis, especially by RNA polymerase II, as the main source of R-loops in the mature sperm. The finding of common enriched motifs in human and bonobo R-loops may primarily reflect the expected similarity of transcriptional patterns in closely related primate species and global transcription in the early stages of spermatogenesis (Xia et al., 2020). On the other hand, the other 39.19% ± 0.81% of the genome wide R-loops show no corresponding transcripts in the mature sperm head, suggesting their presence in non-transcribed regions or non-polyadenylated RNA as well. The applied RNAseq protocols are based on an enrichment of poly-A RNA, but the exact comparison of sperm head transcriptome data in a meaningful quantifiable approach to estimate the relative proportion of sequenced polyadenylated transcripts *versus* non-polyadenylated RNA is pending.

### 3.2 R-loop formation is facilitated in gene bodies

**FIGURE 3:**
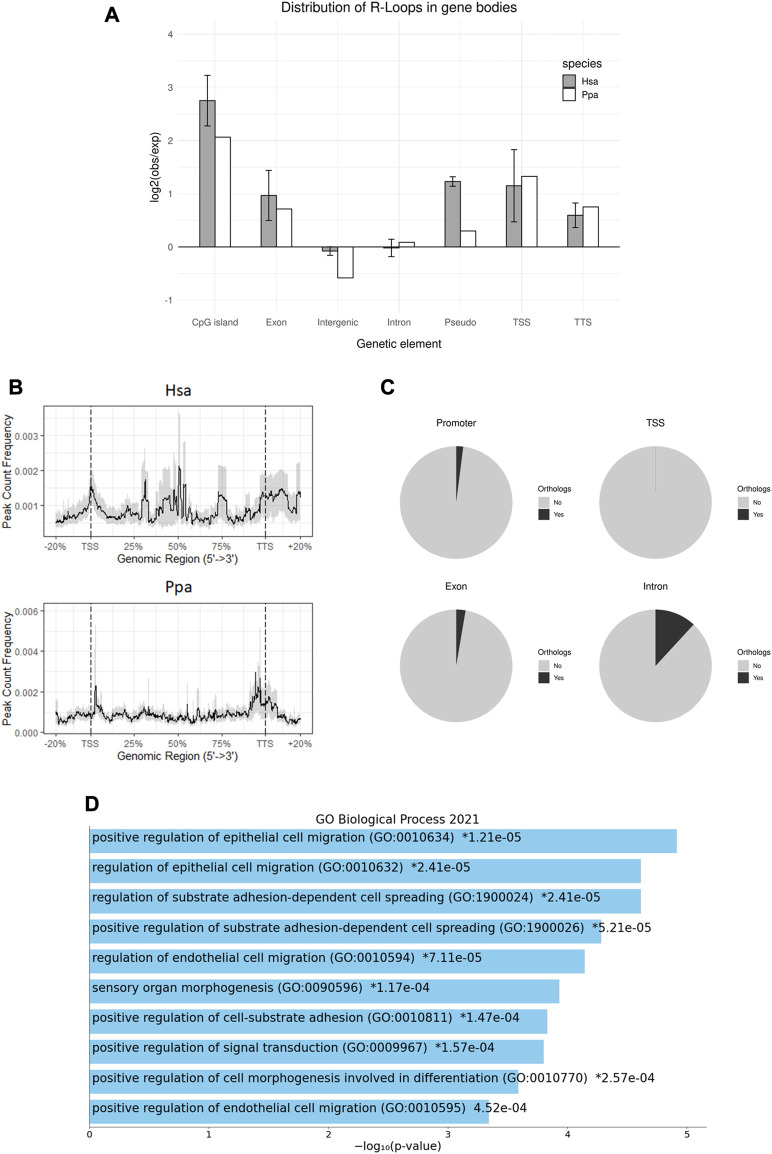
R-loop Distribution across gene bodies. **(A)** Genomic distribution of R-loop peaks across genomic annotations compared to the expected genomic distribution in human and bonobo sperm. Error bars shows ±SEM among the biological replicates. **(B)** Metaplot of the R-loop distribution in human and bonobo sperm across gene bodies, relative to transcription start site and transcription termination site. **(C)** Pie charts showing the proportion of R-loops in in human sperm with an orthologous gene feature in bonobo. **(D)** Summary of GO Biological Process 2021 enrichment results of conserved introns between human and bonobo, ordered by *p*-values.

By associating peaks with genomic features including different classes of TEs on a multilocus level, we found an enriched (log2 (observed/expected)) R-loop formation in gene bodies (Figures 3A,B), as can be observed in somatic cells, too (Sanz et al., 2016; Chen et al., 2017). Owing to their potentially different modes of biogenesis and regulatory effects, the R-loop peaks were further differentiated according to frequencies of their occurrence along the gene body (Figure 3A). Thus, we examined R-loops coinciding with transcription start sites (TSS), exons, introns, and transcription termination sites (TTS). As a result, R-loop formation in human sperm heads was in general strongly favored in TSS, exons and TTS particularly in genes showing a strong GC skew, whereas introns and intergenic regions showed no enrichment in R-loop formation. The highest enrichment was observed for CpG islands (CGI). Similar to human sperm, R-loops in bonobo sperm heads also tend to form in genes, especially in TSS, exons, and TTS, but also in CGIs albeit to a lesser extent as compared to the human situation (Figures 2A, 3A).

### 3.3 Only a minor fraction of R-loops localizes to human-bonobo orthologous loci

To examine a possible locus-specific conservation of R-loops, we pairwise compared single loci for humans with the orthologue in bonobos for conserved R-loop formation. To this end we subdivided the loci under scrutiny into the abovementioned gene components and regulatory regions. Neither in TSS, nor within exons or TTS a significant number of orthologs being identical between humans and bonobos could be seen. Contrasting to this, 10% of the human introns with R-loops shared an intronic R-loop orthologue in the bonobo (Figure 3C). Interestingly, Gene Ontology (GO) enrichment analysis of the corresponding genes using Enrichr (Kuleshov et al., 2016; Chen et al., 2013; Xie et al., 2021) displayed enriched GO terms including positive regulation of epithelial cell migration, positive regulation of substrate adhesion-dependent cell spreading, positive regulation of cell-substrate adhesion and positive regulation of cell morphogenesis involved in differentiation. These biological processes could all be relevant for the early development of a zygote in both species (Figure 3D). In summary, the picture emerges that potentially regulatory R-loops act in a probabilistic manner rather than as discrete epigenetic character states at defined orthologous loci (Adrian-Kalchhauser et al., 2020).

### 3.4 Transposable elements in introns might represent hotspots of R-loop formation

**FIGURE 4:**
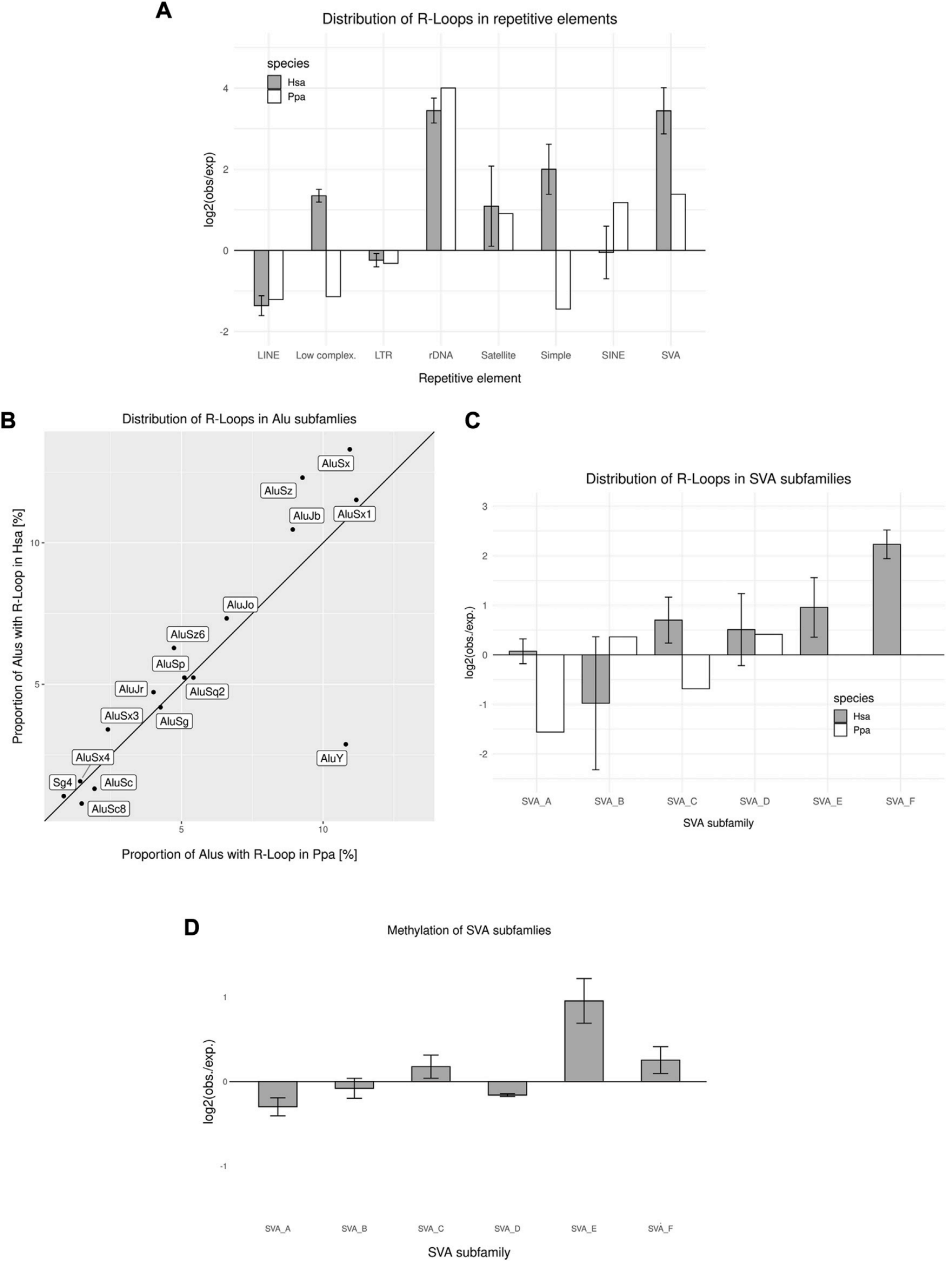
R-loop Distribution across transposable elements. **(A)** Genomic distribution of R-loop peaks across repetitive elements compared to the expected genomic distribution in human and bonobo sperm. **(B)** Distribution of R-loops in different ALU subfamilies in human and bonobo sperm. The bisection indicates an equal distribution between human and bonobo **(C)** Distribution of R-loop peaks in SVA subfamilies compared to the expected distribution in human and bonobo sperm. **(D)** Relative methylation rate of SVA subfamilies. Data in **(A), (C)** and **(D)** represent mean ± SEM among the biological replicates.

TEs constitute a major part of primate genomes, and thus, intronic sequences. With the likely detrimental effect of insertional mutagenesis into exonic sequences, reduced evolutionary constraints hold for introns, although TEs shape the function of their corresponding introns by triggering differential splicing, premature stop codons and maintaining an open chromatin state (Cordaux and Batzer, 2009; Zhang et al., 2011; Ohtani and Iwasaki, 2021). Therefore, in particular larger TEs such as composite retroposons, for example, SVAs, and LINEs are more likely to be conserved in introns and intergenic regions as compared to TE insertions in coding sequences. Moreover, due to the high GC content of primate-specific SINEs and their copy number, they are likely to facilitate the formation of R-loops during transcription at one site and potentially also the re-hybridization of abundant SINE transcripts, respectively (Zeng et al., 2021). R-loops were enriched in rRNA genes, satellite DNA and SVAs, whereas they were underrepresented in LINEs and long terminal repeat retrotransposon (LTRs) (Figure 4A). Therefore, we analyzed two actively transposing and thus transcribed TEs in more detail and looked at hominoid ALUs and SVAs.

Interestingly, the enrichment of SINEs concomitant with R-loops is different in humans and bonobos (Figure 4A). Therefore, we analyzed the proportion of ALU-subfamilies contributing to the R-loop landscape. Most of the ALU-subfamilies showed similar association with R-loops in both human and bonobo sperm. Interestingly, the older subfamilies ALUSz, ALUSx and ALUJb, showed human-specific enrichment, whereas the much younger ALUY (Kapitonov and Jurka, 1996), showed a strong enrichment in bonobo sperm (Figure 4B). Upon comparing the respective TE-R-loop coincidence in both human and bonobo sperm, we observed that in both taxa SVAs were enriched with R-loops. Strikingly, the human-specific families SVA_E and SVA_F, showed the highest R-loop formation rate, whereas the youngest common SVA_D was most enriched in the bonobo (Figure 4C). Compared with bonobo sperm, where all R-loop-covered SVAs were intergenic, 60% of human SVAs associated with R-loops were found in introns. The SVAs in introns were mostly human-specific integrations (81%), resulting in a human-specific signature of intronic R-loop formation in SVAs.

Previous studies have described the absence of methylation at CGI associated with R-loops and protection from *de novo* methylation by DNMT3B1 during early development. Interestingly, some TEs tend to evade the re-methylation during spermatogenesis after a genome wide erasure of epigenetic marks (Molaro et al., 2011; Ginno et al., 2012; Rodriguez-Terrones and Torres-Padilla, 2018; Dietmann et al., 2020). During spermatogenesis nearly the whole genome is transcribed in an extreme global transcription, including TEs (Xia et al., 2020). Therefore, some highly transcribed TEs might adopt R-loops through excessive transcription and their composition thus escaping a subsequent re-methylation through their RNA:DNA hybrid structure. Dietmann et al. (2020) stated that most of the young SVAs stay hypomethylated in human primordial germ cells, after an erasure of epigenetic marks and subsequent re-methylation. Therefore, we compared our DRIP data to sperm methylomes (Molaro et al., 2011). Noticeably, more than 60% of SVAs adopting an R-loop structure are hypomethylated compared to a genome-wide 40% of SVAs without a R-loop association. The human-specific, actively transposing SVA_E and SVA_F are less methylated than the evolutionary old SVAs common to all homininae, with the exception of SVA_C (Figure 4D). These findings of hypomethylated young SVAs in sperm correlate with the results of Dietmann et al. (2020) analyzing human primordial germ cells. Escaping the re-methylation, similar to R-loop associated CGIs, could promote transcription of the hypomethylated SVAs and thus enhance the mobility of young SVAs in the male germline. Moreover, the hypomethylation of intronic SVAs could facilitate transcription in corresponding genes, creating a co-transcriptional influence on genes.

### 3.5 Only a small fraction of sperm head R-loops coincides with residual histones

**FIGURE 5:**
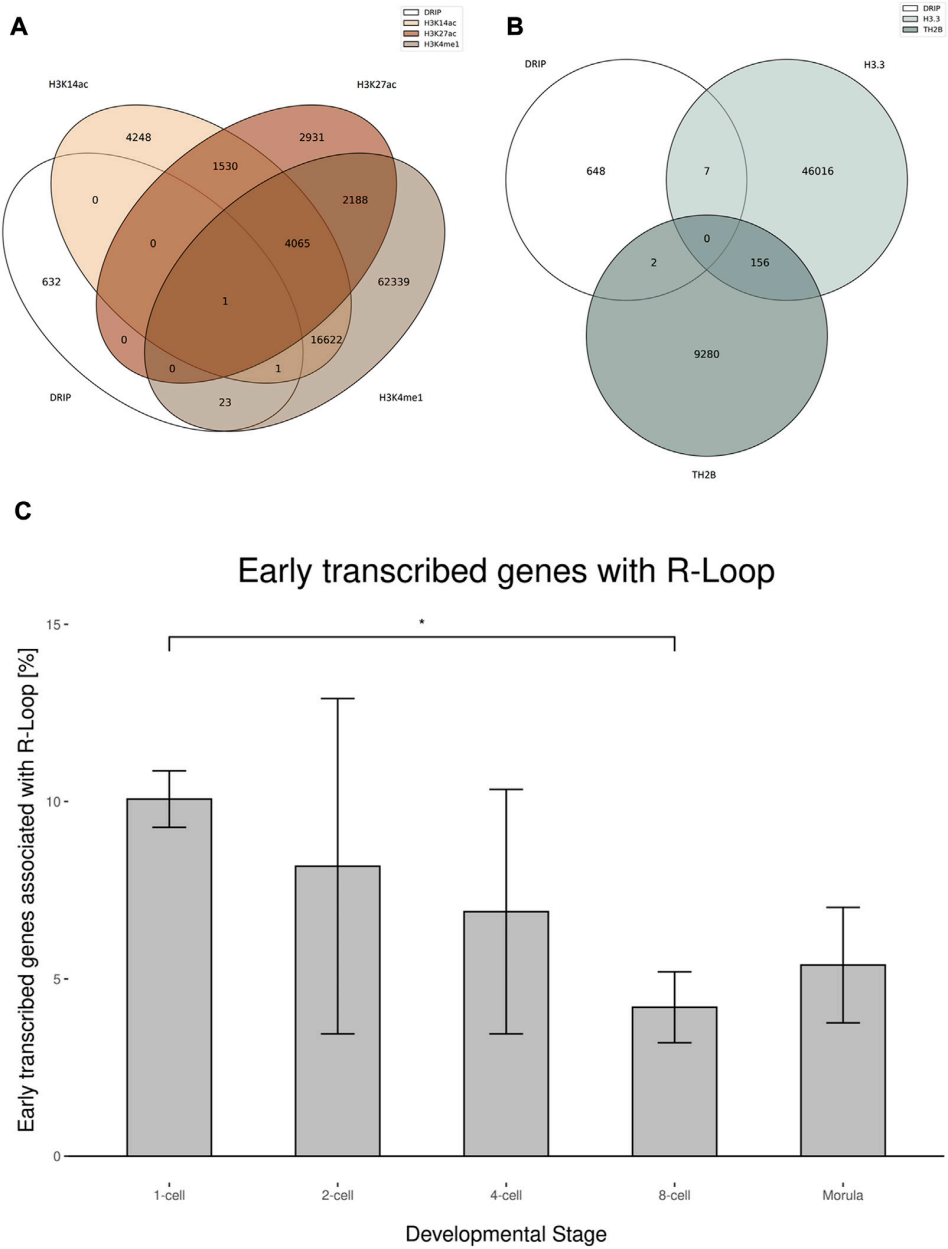
Potential epigenetic effective R-loops in sperm **(A)** Venn diagrams showing the overlap between R-loops and residual histones H3K14ac, H3K4me1 and H3K27ac in human sperm. The overlapping peaks of DRIP replicates were used. **(B)** Venn diagrams showing the overlap between R-loops and residual histones H3.3 and TH2B in human sperm. The overlapping peaks of DRIP replicates was used. **(C)** Fraction of early transcribed genes in the human zygote by developmental stage associated with R-loops in human sperm. Error bars shows ±SEM among the biological replicates. Two-tailed Student’s t-test was used to test for significance (*p* < 0.05).

During spermiogenesis and beginning with the elongating spermatid stage, human sperm chromatin undergoes a dramatic transition in which histones are largely replaced by protamines. Residual histones in spermatogenesis remain because of an incomplete erasure of somatic and transitional histones, such as H3.3 and TH2B. It is hypothesized that they play a potential role in epigenetic inheritance due to their impact on the chromatin state (Wang et al., 2019). In contrast to the tight protamine packaging, residual histones create open chromatin poised for transcription and facilitating a re-hybridization of transcripts and genomic DNA. Therefore, we investigated the coincidence of R-loops in regions with residual histones H3.3 and THB2 from spermatogenesis and residual somatic histones, typically marking open chromatin for active transcription. Previous studies described genomic regions tagged by transcriptionally active chromatin states with the occurrence of H3K27ac, H3K14ac and H3K4me1. Interestingly, no significant overlap was detected between R-loops and marks of transcriptional activity with the above-mentioned modified histones. We further investigated the combined occurrence of H3K27ac and H3K4me1, which marks an active enhancer, due to their interaction with various lncRNAs. Marks of active enhancers also showed no significant correlation with R-loops (Figure 5A). Genomic regions with an incomplete histone-to-protamine transition, retaining the residual histones H3.3 and TH2B, showed no significant overlap with R-loops as well (Figure 5B).

Although residual histones, regardless of their origin during spermatogenesis, should facilitate the formation of R-loops because of potential transcription and an open chromatin state (Hammoud et al., 2011; Schagdarsurengin et al., 2012; Wang et al., 2019), only a fraction of R-loops can be found in regions of residual histones. This leads to the hypothesis that R-loops occur even in the tightly protamine-packed chromatin of sperm or in regions depleted from both protamines and residual histones.

### 3.6 R-loop containing genes might trigger intergenerationally effective epigenetic marks

Like canonical histone modifications, R-loops can influence DNA-protein-interactions, and reconstruct the spatial epigenetic landscape due to its three-dimensional structure (Al-Hadid and Yang, 2016). This global spatial rearrangement of sperm chromatin could potentially play a major role in early transcription and DNA interactions in the pronucleus of the zygote, also implementing an intergenerational epigenetic effect mediated by R-loops. Before the major wave of autonomous transcription during ZGA beginning at the 4-cell-stage of the human zygote, minor transcriptional activities can be detected as early as the formation of the pronuclei directly after fertilization (Vassena et al., 2011; Xue et al., 2013; Rodriguez-Terrones and Torres-Padilla, 2018). This early transcription is assumed to be strongly influenced by epigenetic factors of the sperm and oocyte. In particular, transcription in the male pronucleus would essentially be dependent on the paternal transferred epigenetic state of the sperm genome. This could lead to a male influence on the early zygotic transcription pattern just hours after fertilization, influencing major developmental decisions (Fraser and Lin, 2016).

To correlate the stage specific transcription patterns following zygote formation with our sperm head R-loop data, we compared the presence of R-loops in different genes with their corresponding stage-dependent expression levels as outlined in Xue et al. (2013). Remarkably, the transcription of genes associated with R-loops tends to start early in the zygote, especially directly after fertilization in the 1-cell and 2-cell stages, whereas genes expressed days after fertilization show just few R-loops (Figure 5C). The transcription patterns of the 1-cell and 2-cell stages showed the highest similarity to the sperm R-loop profile, descending to the ZGA and following the autonomous 8-cell stage and morula. The overlap of R-loop-associated genes and oocyte-specific expressed genes was comparable to that of Morula–expressed genes. This trend indicates a strong impact of the R-loop profile to the epigenetic payload of the sperm.

## 4 Discussion

### 4.1 R-loops form during transcription in spermatogenesis

In the present study, we unveiled the presence of R-loops in mature sperm of humans and bonobo. In a genome-wide read out from DRIP-Seq experiments, we observed a strong correlation between R-loop abundance and gene density regarding chromosomes. The R-loops present in both primates show a GC bias and an enrichment in gene bodies, particularly in protein-coding genes. Most of the R-loop associated genes even show a strong GC skew. We observed evolutionary conserved R-Loops in orthologous introns of human and bonobo corresponding to potentially early developmental processes. The motif enrichment analysis revealed conserved motifs in R-loops of both taxa. The most enriched motifs are binding sites for TF of RNA polymerase II, which primarily transcribes mRNAs and miRNAs. Furthermore, more than half of the human R-loops show a corresponding transcript in the transcriptome of the mature sperm, which results from transcription during early spermatogenesis, because of a mostly inert transcription in sperm (D’Occhio et al., 2007; Rathke et al., 2007). These findings implicate the virtually genome-wide transcription during spermatogenesis as the main contributor to the sperm R-loop landscape. Moreover, R-loop formation as a byproduct of transcription is favored by GC skew of transcribed genes. Both the transcription-coupled R-loop formation leading to an R-loop pattern and the fact that R-loop RNA oligonucleotides constitute a significant part of the non-coding RNA population in sperm remind them of somatic cells.

### 4.2 TEs as evolutionary hotspots for R-loop formation

Hypothetically and because of their GC skew and repetitive nature, TEs could function as species-specific anchors for R-loops across evolutionary time scales (Zeng et al., 2021). The primate-specific ALUs and SVAs show both species-specific and evolutionary conserved trends with respect to the associated R-loops. Overall ALUs associated with R-loops were enriched in bonobo but not in human sperm. We observed a very similar distribution of R-loops in most of the ALU-subfamilies, whereas older Subfamilies ALUSz, ALUSx and ALUJb show an enrichment bias towards human sperm. Interestingly, the youngest ALUY in association with R-loops shows a strong enrichment in bonobo. This association causes a genome-wide enrichment of ALUs covered in R-loops and therefore a species-specific feature. The SVA is of ever-increasing interest due to its co-evolution with TF and thus the potential regulation of expression in nearby genes (Savage et al., 2013; Barnada et al., 2022). We detected an enrichment of SVAs associated with R-loops as a conserved feature in both human and bonobo sperm. Interestingly, the human-specific SVA_F, which is primarily located in introns, showed the strongest enrichment in R-loops. The humans-specific SVA_E and SVA_F are involved in genomic changes during recent human evolution (Cordaux and Batzer, 2009; Gianfrancesco et al., 2019). The SVA_F-subfamily, the youngest and still actively transposing in the human genome, even produced recent fusions with exons and CGIs of multiple genes (Gianfrancesco et al., 2019). These characteristics make SVA_F an evolutionary interesting human-specific feature in R-loops of mature sperm. Interestingly, our data suggests that the R-loop covered SVAs tend to remain hypomethylated, which could well influence transcription and the epigenetic state of adjacent genes during early embryonal development, and therefore function as cis-regulatory elements comparable to previous described findings of co-transcription of genes in human stem cells (Barnada et al., 2022). We detected transcripts of each SVA-subfamily in the transcriptome of mature sperm, validating their expression during spermatogenesis. The transcription of the TEs is mainly suppressed during spermatogenesis by a multitude of mechanism (Reznik et al., 2019), although some TEs can still be mobilized causing new integrations. We speculate that actively transposing SVAs could theoretically also be suppressed by the formation of regional R-loops after global demethylation and before the PIWI-piRNA-system is effective, highlighting the double-edged task of fostering gene transcription during gametogenesis without mobilizing TEs (Dietmann et al., 2020). Until now, only few data on the quantitative and qualitative aspects of the association between SVA and R-loops are published. To support our conclusion of SVA silencing by R-loops, we exploited the link between H3S10 phosphorylation and R-loops as a proxy to check H3S10p-ChIP data from human IMR-5 cells for the possible enrichment of SVA sequences (Castellano-Pozo et al., 2013; Roeschert et al., 2021). As a result, we obtained a slight enrichment of SVAs in general and more specifically an enrichment of the youngest human-specific SVA_F in somatic cells. This pattern of H3S10p enrichment of SVA sequences in somatic cells, though to a lesser extent, is strikingly similar to what we detected in sperm heads for the R-loop-SVA intersection thus supporting the hypothesis of R-loops promoting the silencing of SVAs from a somatic perspective.

### 4.3 R-loops as epigenetic marks

During spermatogenesis the histone-to-protamine transition ensures the integrity of the paternal DNA in the spermhead. An incomplete histone-to-protamine transition leaves genomic regions associated with residual histones, leading to an open chromatin state, poised to DNA-RNA interactions (Patankar et al., 2021). We differentiated between residual histones in marks of active transcription, like H3K14ac, H3K4me1 and H3K27ac and spermatogenesis relevant histones H3.3 and TH2B. Interestingly we observed neither a significant overlap of H3K14ac, H3K4me1 and H3K27ac with R-loops nor with H3.3 and TH2B. Previous studies hypothesized residual histones as an important paternal epigenetic contribution to the early transcription of the zygote (Torres-Flores and Hernández-Hernández, 2020; Patankar et al., 2021). Similarly, R-loops are like canonical histone modifications-able to influence DNA-protein-interactions, and can reconstruct the spatial epigenetic landscape (Al-Hadid and Yang, 2016). Moreover, the three-stranded nature of R-loops affects the broader chromatin confirmation of the sperm genome and could thus play a locally antagonistic role to the tight protamine packaging. Therefore, we investigated the correlation between R-loop associated genes in mature sperm and paternal influenced early transcription before the zygotic genome activation. From these analyses we conclude that genes which are transcribed in the 1- and 2-cell stadium of the zygote tend to be associated with R-loops in mature sperm. This association decreases in later developmental stages closer to the zygotic genome activation and the final autonomous transcription in the 8-cell stage (Zhang et al., 2009; Vassena et al., 2011). We hypothesize that these R-loops facilitate interaction between DNA and the transcriptional machinery through an open chromatin state and therefore play an important role in fine tuning the early transcription in the zygote, particularly in the male pronucleus. Although, we have provided evidence for the male side of the regulatory R-loop landscape, it will be important to perform similar and infraspecific comparative NGS experiments for the female side. In this way the early zygotic R-loop landscape can be disentangled regarding the relative contribution of paternal and maternal germ cell R-loops to the zygote. *In vitro* gametogenesis starting from pluripotent stem cells may be an obvious strategy to complement sperm with oocyte data and to characterize the role of regulatory R-loops in epigenetic inheritance more completely (Saitou and Hayashi, 2021). Finally, we propose a new intergenerationally effective epigenetic mechanism influencing expression in the early embryo through paternally transmitted R-loops. Taken together, R-loops, the non-coding RNAs that constitute them, and the respective consequences on chromatin structure should be added to the spectrum of the sperm cell’s payload to the zygote.

## Data Availability

Original datasets are publicly available in SRA and can be found here: PRJNA890147. Publicly available datasets were analyzed in this study. The names of the repository/repositories and accession number(s) can be found in the article/supplementary material.
